# Recurrent episodes of intractable laryngospasm followed by laryngeal and pulmonary oedema during dissociative anaesthesia with intravenous ketamine

**DOI:** 10.4103/0019-5049.68395

**Published:** 2010

**Authors:** Neha Baduni, Manoj Kumar Sanwal, Aruna Jain, Nisha Kachru

**Affiliations:** Department of Anaesthesiology and Intensive Care, Lady Hardinge Medical College & Associated Hospitals, Shaheed Bhagat Singh Marg, New Delhi - 110 001, India

Sir,

Ketamine is commonly used to provide analgesia, amnesia and sedation for painful pediatric procedures. Its most dangerous side effects include: laryngospasm and apneic spells.[1] We report a case of recurrent intractable laryngospasm, followed by laryngeal and pulmonary oedema with intravenous ketamine.

A seven-year-old, 16 kg, ASA grade I child was admitted for change of Plaster of Paris cast. One week earlier he had undergone a posterior medial soft tissue release surgery for neglected CTEV, the perioperative period being uneventful. After securing an intravenous access he was administered inj. midazolam 1 mg, inj. ketamine 15 mg, inj. glycopyrrolate 0.16 mg along with supplemental oxygen. He went into laryngospasm even before the procedure was started, which was broken easily with positive pressure ventilation (PPV) with 100% O_2_. The rest of the intraoperative period was uneventful. But as soon as the child was shifted on transport trolley, he went into laryngospasm again which was broken by succinylcholine (5 mg) along with PPV. As soon as the effect of succinylcholine wore off, the child went into laryngospasm for the third time. This episode resolved with forward displacement of mandible and PPV. An oral suctioning was done to remove any collected secretions along with intravenous lidocaine. Within the next few minutes, he went into laryngospasm again. We decided to intubate the trachea after giving propofol and succinylcholine. The child was extubated only when fully awake. Shockingly, he went into complete laryngospasm, again, with falling SpO_2_, for the fifth time. Auscultation now revealed fine crepitations all over the chest. Fearing negative pressure pulmonary oedema, he was intubated and given lasix (10 mg) and morphine (2.0 mg). Direct laryngoscopy showed an oedematous epiglottis and vocal cords. He was given adrenaline 0.25 mg diluted in normal saline intravenously along with dexamethasone 2 mg iv. Crepitations disappeared within the next 10 minutes. A repeat direct laryngoscopy revealed a reduction in the epiglottic oedema. IV lidocaine 20 mg and nebulisation with adrenaline were given. The trachea was extubated after gentle suctioning. He was shifted to PACU where subsequent follow ups for the next 24 hours did not reveal any fresh episode of desaturation.

The incidence of laryngospasm in children undergoing general anaesthesia has been reported to be between 0.78% and 5%.[2] Common precipitating factors include insufficient depth of anaesthesia on intubation or extubation, or the presence of an airway irritant such as blood, mucus, surgical debris or any foreign body, prior history of active respiratory tract infection, allergies, airway anomaly or pre existing gastro oesophageal reflux disease. In our patient, pre operative assessment ruled out any pre-existing factors. Though the movement of the patient while shifting could have precipitated laryngospasm.

Failure to treat laryngospasm in a timely manner is dangerous. In addition to hypoxemia, some patients who are able to generate very large negative inspiratory pressures, when attempting to breathe against the obstruction, may develop negative pressure pulmonary oedema. There are several reports of the occurrence of laryngospasm and apnea after the use of ketamine, but they are more with intramuscular rather than intravenous use.[3] Also, in most of these cases the episodes were transient and responded to airway alignment maneuvers, supplemental oxygen or brief PPV. There is only one case report of recurrent laryngospasm after the use of intramuscular ketamine sedation in children.[4] But this was self resolving, unlike ours which demanded aggressive therapy. Ours is probably the first case report of repeated intractable episodes of laryngospasm after intravenous ketamine.

Even though ketamine has a wide safety margin, rare but serious side effects are always to be kept in mind and managed aggressively.

## References

[1] Smith JA, Santer LJ (1993). Respiratory arrest following intramuscular ketamine injection in a 4 year old child. Ann Emerg Med.

[2] Olsson GL, Hallen B (1984). Laryngospasm during anaesthesia. A computer-aided incidence study in 136, 929 patients. Acta Anaesthesiol Scand.

[3] Elliot M, Bachur R (2009). Serious adverse events during procedural sedation with ketamine. Ped Em Care.

[4] Cohen VG, Krauss B (2006). Recurrent episodes of intractable laryngospasm during dissociative sedation with intramuscular ketamine. Ped Em Care.

